# Regioselective Approach to Characterizing Increased Edge Availability in Layered Crystal Materials following Layer Expansion: Reaction of Kaolinite with Octadecyltrimethylammonium Salts

**DOI:** 10.3390/ma15020588

**Published:** 2022-01-13

**Authors:** Shingo Machida, Ken-ichi Katsumata, Atsuo Yasumori

**Affiliations:** Department of Material Science and Technology, Faculty of Advanced Engineering, Tokyo University of Science, 6-3-1 Niijuku, Katsushika-ku, Tokyo 125-8585, Japan; k.katsumata@rs.tus.ac.jp (K.-i.K.); yasumori@rs.tus.ac.jp (A.Y.)

**Keywords:** kaolinite, edge modification, alkylammonium ion, organically-modified kaolinite, intercalation

## Abstract

In this paper, the regioselective reactions of kaolinite and methoxy-modified kaolinite (MeO-Kaol), methanol-expanded kaolinite, with octadecyltrimethylammonium salts are compared. This study mainly concerns the reactions of kaolinite or MeO-Kaol with octadecyltrimethylammonium chloride (C18TAC) in methanol and the subsequent exhaustive washing of the resultant products with ethanol. X-ray diffraction patterns of the products reveal no intercalation of C18TAC between pristine kaolinite layers. Additionally, intercalation and subsequent deintercalation of C18TAC proceed in the product using MeO-Kaol. In the Fourier-transform infrared spectra, the intensities of CH_2_ stretching bands of the product prepared using MeO-Kaol drastically increase compared to those using kaolinite. In addition, CH_2_ stretching bands of the product using kaolinite are hardly observed without enlarging the spectrum. The product using MeO-Kaol also displays mass loss in the range of 200–300 °C in the thermogravimetric curve and a nitrogen content with 0.15 mass% estimated using the CHN analysis. These results therefore demonstrate an increase in the available reactive edges in the layered crystal material following an expansion of the stacked layers.

## 1. Introduction

Layered crystal materials comprise stacked inorganic layers, each of which possesses both layer and edge surfaces. The latter can result from crystal fractures and exhibit different reactivities to the former [1,2,3,4,5,6]. The access of ions/molecules to these edge sites can be increased by the intercalation of guest species, which expands the spacing between the stacked layers [7,8,9,10]. Therefore, an increase in these edge sites following intercalation can provide evidence that the mechanism involves edge reactivity. However, interactions of ions/molecules with edge surfaces are generally the same as those with layered ones.

Herein, we report the reactions of kaolinite, a regioselective layered crystal material, with octadecyltrimethylammonium salts. Kaolinite is a layered aluminosilicate having the formula Al_2_Si_2_O_5_(OH)_4_, and is made of stacked asymmetrical layers, each of which consists of an AlO_2_(OH)_4_ sheet and a SiO_4_ sheet. These neutral layers allow the intercalation of salts or neutral molecules bearing polar groups that are readily deintercalated upon washing with appropriate solvents [9,10]. In contrast, the kaolinite edges are crystal fracture surfaces at which cation-exchange sites are present [1,11]. Changes in edge availability depending on the degree of layer expansion can be assessed by comparing pristine kaolinite with kaolinite intercalation compounds in which no layer shrinkage during reactions is preferable. Methoxy-modified kaolinite (hereafter denoted as MeO-Kaol), organically-modified kaolinite with the formula Al_2_Si_2_O_5_(OH)_4_-x(OCH_3_)x (where x never exceeds 1) [12], is a helpful compound for such studies because this material is able to accommodate water molecules in a stable manner under ambient conditions [13]. MeO-Kaol is a type of kaolinite hydrate [14,15,16,17] and can exhibit a 0.86 nm basal spacing (hereafter referred to as 0.86-nm hydrate). MeO-Kaol can also be swollen by methanol molecules [13]. The present research compared the reactions of kaolinite and MeO-Kaol with octadecyltrimethylammonium chloride (C18TAC) and bromide (C18TAB). In a previous study, alkyltrimethylammonium chlorides having relatively long alkyl chains (CnH_2_n + 1N(CH_3_)_3_Cl; n = 16, 18) were successfully intercalated into MeO-Kaol, while the intercalation of hexadecyltrimethylammonium bromide (C16TAB) was not successful [18]. This previous study also showed that the intercalation of hexadecyltrimethylammonium chloride (C16TAC) into kaolinite with concurrent exfoliation of its layers results in the conversion of the original hexagonal plate morphology into nanoscrolls, while the intercalation of C18TAC does not have this effect [18].

## 2. Materials and Methods

The kaolinite used in this study was well-crystallized KGa-1b (i.e., Georgia kaolin) obtained from the Source Clays Repository of the Clay Material Society (Chantilly, VA, USA). MeO-Kaol was prepared according to a previously published method [13]. The reactions of kaolinite or MeO-Kaol with C18TAC or C18TAB were conducted in a similar manner to the reactions in a prior study [18] to give products designated herein as C18TAC/MeO-Kaol, C18TAB/MeO-Kaol, and C18TAC/kaolinite. Following each reaction, the solid products were washed with ethanol and then dried at 80 °C for an hour to give materials referred to herein as C18TAC/MeO-Kaol_Wash, C18TAB/MeO-Kaol_Wash, and C18TAC/kaolinite_Wash. X-ray diffraction (XRD) patterns (XRD-6100, Shimadzu, Kyoto, Japan) were acquired to assess the degree of layer expansion in kaolinites, while Fourier-transform infrared (IR) spectroscopy (FT-IR 4100, JASCO, Ishikawa, Japan) and thermogravimetry (TG; DTG-60, Shimazu, Kyoto, Japan) were used to determine the presence of alkyl chains in kaolinite and the washing products. In order to detect carbon and nitrogen in the specimen using MeO-Kaol and C18TAC with exhaustive washing, CHN analysis was conducted (2400ІІCHNS/O, Perkin Elmer, Waltham, MA, USA). The conformation of alkyl chains in this solid was determined using IR and solid-state ^13^C nuclear magnetic resonance (NMR) spectra with cross polarization (CP) and magic angle spinning (MAS) techniques (ECA-400, JEOL, Tokyo, Japan). Field-emission scanning electron microscopy (FE-SEM) was used to assess the particle morphologies of the products (spra40, Zeiss, Oberkochen, Germany).

## 3. Results and Discussion

**Figure 1:**
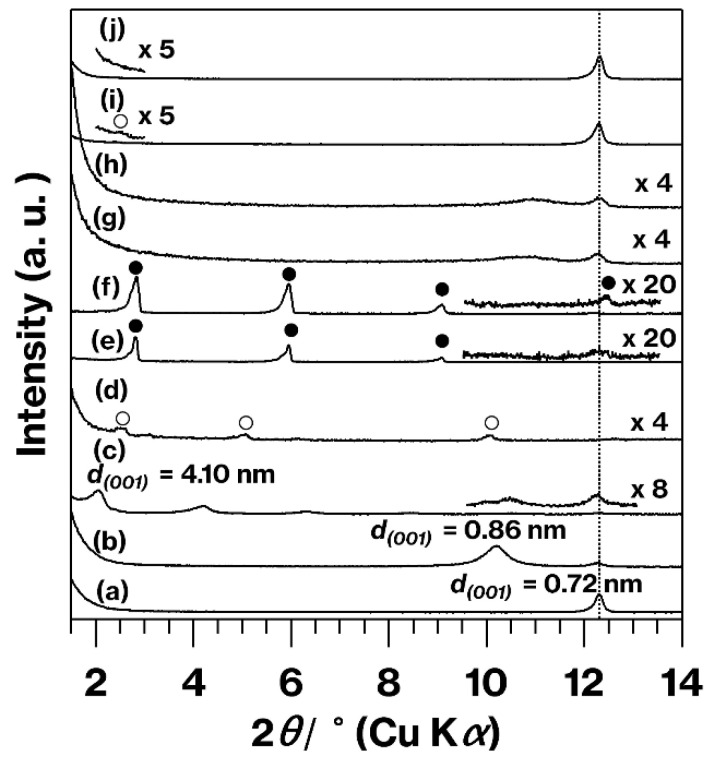
XRD patterns obtained from (a) kaolinite, (b) MeO-Kaol, (c) C18TAC/MeO-Kaol, (d) C18TAC, (e) C18TAB/MeO-Kaol, (f) C18TAB, (g) C18TAC/MeO-Kaol_Wash, (h) C18TAB/MeO-Kaol_Wash, (i) C18TAC/kaolinite, and (j) C18TAC/kaolinite_Wash. Reflections due to C18TAC and C18TAB are marked as open and closed circles, respectively. For (c), (e), (f), (i), and (j), adequately enlarged patterns in some 2*θ* ranges are shown above each original pattern.

Figure 1 presents the XRD patterns for kaolinite and the products. It is evident that reflections due to the original kaolinite layers with a basal spacing of 0.72 nm (the (001) plane [14]) remain in the patterns for the products as a result of the incomplete intercalation capability of kaolinite, which is commonly observed [19]. The MeO-Kaol pattern (Figure 1(b)) is consistent with that obtained in a previous study [20], and the *d* value of 4.10 nm in the C18TAC/MeO-Kaol pattern (Figure 1(c)) is the same as that found in a previous study [18]. The C18TAB/MeO-Kaol pattern (Figure 1(e)) contains only diffraction lines due to C18TAB (Figure 1(f)) at 2*θ* values smaller than the value for the 0.86 nm diffraction line due to MeO-Kaol (2*θ* = 10.3). This result indicates that there was no intercalation of C18TAB between the layers of MeO-Kaol, in agreement with a previous report [18]. The XRD pattern obtained from C18TAC/MeO-Kaol_Wash (Figure 1(g)) demonstrates the disappearance of diffraction lines due to C18TAC/MeO-Kaol, as well as the presence of broader diffraction lines in the 2*θ* range of 10–11° associated with the basal spacings of various types of kaolinite hydrates [14,15,16,17]. Thus, these results indicate the deintercalation of C18TAC from the kaolinite layers. The diffraction line observed in the pattern for C18TAB/MeO-Kaol (Figure 1(e)) is absent in that for C18TAB/MeO-Kaol_Wash, which exhibits a broader diffraction line in the 2*θ* range of 10–11° (Figure 1(h)). The intensities of the broader diffraction lines in the range 10–11° relative to the 0.72 nm diffraction lines attributed to kaolinite in the C18TAC/MeO-Kaol_Wash and C18TAB/MeO-Kaol_Wash patterns decrease compared to the relative intensity of the MeO-Kaol (0.86 nm) diffraction line (0.86 nm) to the kaolinite diffraction line (0.72 nm) in the MeO-Kaol pattern (Figure 1(b)). These results are consistent with the deintercalation or unsuccessful intercalation of guest species when using MeO-Kaol [18,20]. In contrast to the kaolinite pattern (Figure 1(a)), the C18TAC/kaolinite pattern (Figure 1(i)) shows a diffraction line that is also present in the C18TAC pattern (Figure 1(d)). In addition, this line is not evident in the C18TAC/kaolinite_Wash pattern (Figure 1(j)).

**Figure 2:**
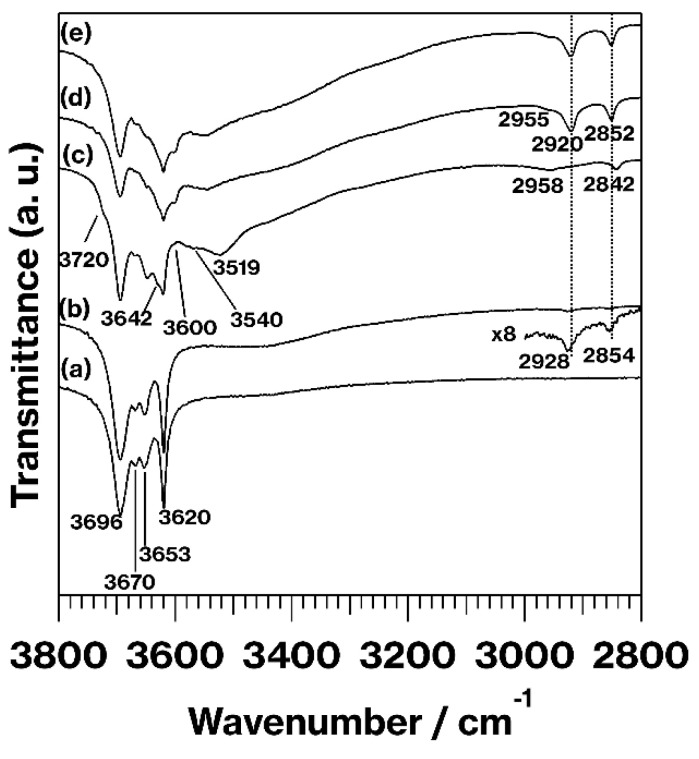
IR spectra in the range of 3800-2800 cm^−1^ obtained from (a) kaolinite, (b) C18TAC/kaolinite_Wash, (c) MeO-Kaol, (d) C18TAC/MeO-Kaol_Wash, and (e) C18TAB/MeO-Kaol_Wash.

Figure 2 presents IR spectra in the range of 3800–2800 cm^−1^ for kaolinite and the products. Regarding the CH stretching region, the C18TAC/kaolinite_Wash spectrum contains weak bands at 2928 and 2854 cm^−1^ (Figure 2(b)) assigned to CH_2_ asymmetrical and symmetrical stretching [21]. The bands in the CH stretching region of the MeO-Kaol spectrum (Figure 2(c)) are similar to those observed in a previous study [13]. The band at 2842 cm^−1^ which appears in the MeO-Kaol spectrum (Figure 2(c)) is absent in the spectra of C18TAC/MeO-Kaol_Wash and C18TAB/MeO-Kaol_Wash. These spectra also show bands at 2920 and 2852 cm^−1^ due to CH_2_ asymmetrical and symmetrical stretching [21] (Figure 2(d,e)), both of which appear at lower wavenumbers compared with those in the C18TAC/kaolinite_Wash spectrum (Figure 2(b)). Additionally, shoulders at 2955 cm^−1^ due to CH stretching of MeO-Kaol [13] and/or CH_3_ stretching [21] appear in the spectra obtained from C18TAC/MeO-Kaol_Wash and C18TAB/MeO-Kaol_Wash (Figure 2(d,e)). Concerning the OH stretching region, the original kaolinite generats four bands attributed to OH stretching at 3969, 3670, and 3653 cm^−1^ (assigned to interlayer hydroxyl groups) and at 3620 cm^−1^ (assigned to inner-layer hydroxyl groups) (Figure 2(a)) [10,22]. Following expansion of the kaolinite layers, the intensities of the bands at 3969, 3670, and 3653 cm^−1^ relative to that at 3620 cm^−1^ decreased, along with the appearance of new OH stretching bands at lower wavenumbers [10,22]. The spectrum of C18TAC/kaolinite_Wash (Figure 2(b)) is also seen to be equivalent to that of kaolinite. The MeO-Kaol spectrum (Figure 2(c)) is a good match for that acquired in a previous study [14]. The spectrum of the MeO-Kaol also provides information regarding 0.86 nm hydrates [14]. Note also that the MeO-Kaol spectrum contains new OH stretching bands at 3720 and 3642 cm^−1^ (assignable to additional interlayer hydroxyl groups [13]), 3600 cm^−1^ (assignable to hydrogen-bonded interlayer hydroxyl groups [20,21,22,23]), and at 3540 and 3519 cm^−1^ (assignable to interlayer water molecules) [13]. However, it should be noted that the evident differences in these five bands could also be related to variations in the degree of hydrogen bonding [13]. The C18TAC/MeO-Kaol_Wash and C18TAB/MeO-Kaol_Wash spectra (Figure 2(d,e)) are slightly different from that of MeO-Kaol (Figure 2(b)), while the intensities of the bands at 3969, 3670, and 3653 cm^−1^ relative to the band at 3620 cm^−1^ decrease compared with the kaolinite spectrum (Figure 2(a)). Additionally, a band at 3540 cm^−1^ is present in the spectra obtained from C18TAC/MeO-Kaol_Wash and C18TAB/MeO-Kaol_Wash (Figure 2(d,e)).

**Figure 3:**
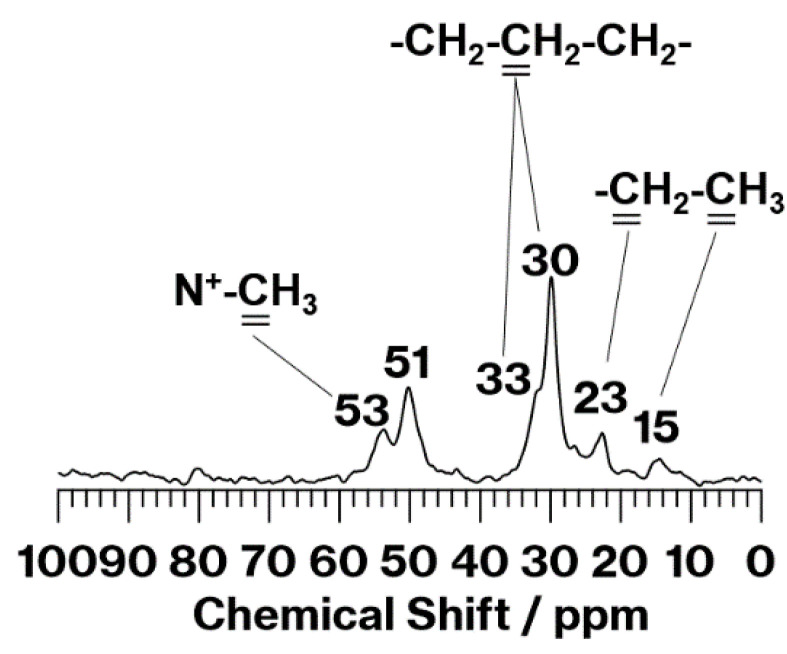
^13^C CP MAS NMR spectrum of C18TAC/MeO-Kaol_Wash.

Figure 3 shows the ^13^C CP MAS NMR spectrum of C18TAC/MeO-Kaol_Wash. This spectrum exhibits signals at 53, 33, 30, 23, and 15 ppm, all of which are assignable to carbons in alkyl chains [24,25]. Among them, the signals at 33 and 30 ppm, which are assignable to the interior methyl carbons, are due to *gauche* and all-*trans* conformations of alkyl chains [24,25], respectively. Additionally, the signal at 51 ppm assigned to methoxy groups [18,23] is present in the spectrum, indicating that methoxy groups remain after the reaction and washing procedure.

**Figure 4:**
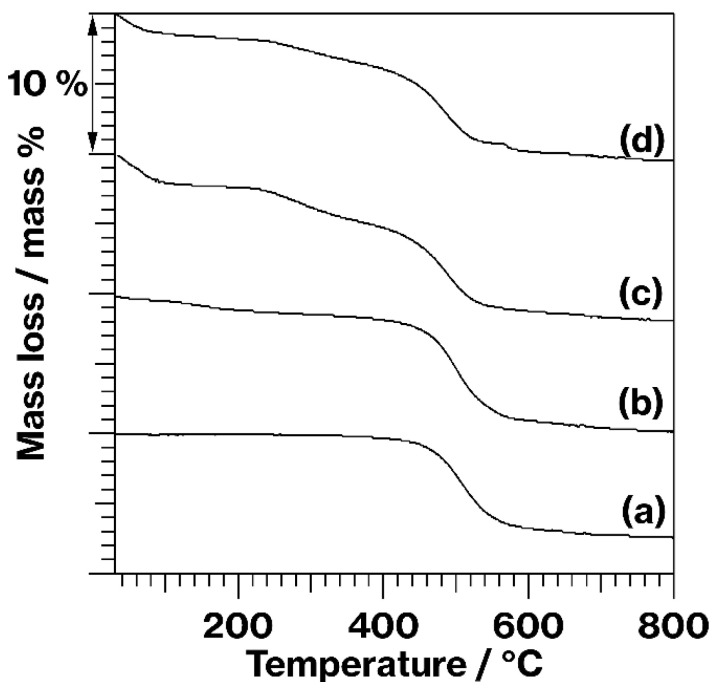
TG curves obtained from (a) kaolinite, (b) MeO-Kaol, (c) C18TAC/MeO-Kaol_Wash, and (d) C18TAB/MeO-Kaol_Wash.

Figure 4 displays TG curves for kaolinite and the products. The curves for kaolinite and MeO-Kaol (Figure 4(a,b)) are similar to those obtained in a previous study [23] and exhibit mass losses in the range of 400–600 °C due to the dehydroxylation of kaolinite [23,26]. This mass loss is also seen in the C18TAC/MeO-Kaol_Wash and C18TAB/MeO-Kaol_Wash curves (Figure 4(c,d)), although at slightly lower temperatures than those for kaolinite and MeO-Kaol (Figure 4(a,b)); the onset temperatures were lower by approximately 50–100 °C. The curves for C18TAC/MeO-Kaol_Wash and C18TAB/MeO-Kaol_Wash also show mass losses at similar onset temperature, within the range of 200–300 °C (Figure 4(c,d)). When C18TAC/MeO-Kaol_Wash representatively underwent CHN analysis, this product contained carbon (3.86 mass%) and nitrogen (0.15 mass%). The mass losses in the range of 200–300 °C were thus likely as a result of the degradation of alkyl chains [26,27]. Additionally, the mass loss in the range of 200–300 °C in the TG curve for C18TAC/MeO-Kaol_Wash is approximately 1 mass% larger than that for C18TAB/MeO-Kaol_Wash, while losses of 2–3 mass% can be observed below 100 °C in the C18TAC/MeO-Kaol_Wash and C18TAB/MeO-Kaol_Wash curves.

It should be noted that the weight ratio of carbon to nitrogen (carbon/nitrogen) of C18TAC is 18 (=12 × 21/14). In contrast, based on the carbon (3.86 mass%) and nitrogen (0.15 mass%) contents of C18TAC/MeO-Kaol_Wash estimated using CHN analysis, the weight ratio of carbon to nitrogen of this product is 25.7 (=3.86/0.15). The difference of these values is due to the presence of methoxy groups in C18TAC/MeO-Kaol_Wash, revealed by the ^13^C CP MAS NMR spectrum (Figure 3).

Note also that previous reports have suggested that the interlayer water in MeO-Kaol results from atmospheric moisture and/or trace amounts of water in the methanol used to process this material [13]. For unexplained reasons, methanol molecules tend to be spontaneously deintercalated from the layers of MeO-Kaol under ambient conditions, while water molecules remain in the interlayers [13]. This fact was acceptable for deintercalation or unsuccessful intercalation of guest species when using MeO-Kaol at room temperature [18,20]. Thus, water molecules tend to remain in the MeO-Kaol interlayers when the material undergoes a reaction under mild conditions. The amounts of interlayer water in kaolinite hydrates are typically in the range of 2–5 mass% depending on the drying conditions [13,23,28], although these conditions are not always reported in detail [13,28].

**Figure 5:**
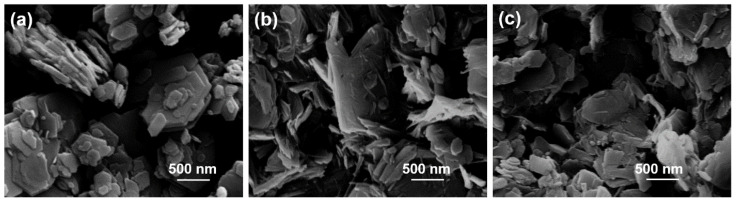
FE-SEM images of (**a**) MeO-Kaol, (**b**) C18TAC/MeO-Kaol_Wash, and (**c**) C18TAB/MeO-Kaol_Wash.

Figure 5 illustrates FE-SEM images of MeO-Kaol, C18TAC/MeO-Kaol_Wash, and C18TAB/MeO-Kaol_Wash. The image of MeO-Kaol (Figure 5a) exhibits the hexagonal plate-like morphology of kaolinite which is similar to those reported previously [23,26]. This morphology is also observed in the images of C18TAC/MeO-Kaol_Wash and C18TAB/MeO-Kaol_Wash, while the hexagonal shapes are slightly ambiguous with the appearance of a portion of rod-like morphology which is similar to those reported in the previous reports [18,26]. Additionally, the extent of the ambiguity of C18TAC/MeO-Kaol_Wash is greater than that of C18TAC/MeO-Kaol_Wash. Note that the presence of rod-like morphology is a result of the rolling up of kaolinite layers. A portion of kaolinite layer exfoliation thus likely occurred in C18TAC/MeO-Kaol_Wash and C18TAB/MeO-Kaol_Wash.

Together, the XRD patterns (Figure 1), IR spectra (Figure 2), and TG curves (Figure 4) indicate that C18TAC/MeO-Kaol_Wash and C18TAB/MeO-Kaol_Wash were kaolinite hydrates with alkyl chains on their outer surfaces. The various hydration states of these materials were evident from their XRD patterns (Figure 1(h,g)). It is known that the hydration states of kaolinite hydrates are affected by various factors, including reaction conditions and the chemical components of the kaolinite layers, for example, following fluorination or methoxylation [13,14,15,16,17]. In addition, the intercalation and deintercalation of guest species, as well as the reactions of guest species with edges, can affect the stacking order of the layers. As a consequence, a portion of the kaolinite layers were rolled up (Figure 5b,c). This is well-consistent with the previous report showing the deintercalation of alkylamines with concurrent exfoliation and rolling up of kaolinite layers [29]. Since the previous report reveals that the C18TAC intercalation cannot induce the rolling up of kaolinite layers [18], the deintercalation of C18TAC likely has the effect based on the greater extent of ambiguous shapes of C18TAC/MeO-Kaol_Wash than C18TAB/MeO-Kaol_Wash (Figure 5b,c) which shows no intercalation of C18TAB. Thus, the difference in morphologies between C18TAC/MeO-Kaol_Wash and C18TAB/MeO-Kaol_Wash is due to the degree of kaolinite layer expansion by guest species before washing with ethanol; kaolinite layers were expanded by C18TAC for C18TAC/MeO-Kaol, whereas those were never expanded by C18TAB for C18TAB/MeO-Kaol. As compared to these products, the present study obtained MeO-Kaol by washing with methanol based on the previous method [13]. Additionally, based on the reflections due to the basal spacing of MeO-Kaol and methanol-intercalated kaolinite shown in previous reports [18,20], the deintercalation of methanol from kaolinite layers does not affect kaolinite morphology. It should be noted that ethanol molecules are hardly intercalated between kaolinite layers, even under high-pressure conditions [30]. Note also that the kaolinite intercalation compounds often accompany with neat liquids and bulk solids [10,19]. This is because kaolinite intercalation reactions proceed when neat liquids or high concentration solutions are used, and guest species of kaolinite intercalation compounds are easily deintercalated upon washing with solvents; thus, kaolinite intercalation compound are generally unwashed [10,19]. Therefore, characteristics of kaolinite intercalation compounds often contains information of neat liquid or bulk solids. Unfortunately, the hexagonal plate-like morphologies of C18TAC/MeO-Kaol and C18TAB/MeO-Kaol are ambiguous in the FE-SEM images since the huge amounts of C18TAC and C18TAB remains, while the rod-like morphology due to nanoscrolls is hardly observed (data not shown). 

The TG data did not show significant differences between C18TAC/MeO-Kaol_Wash and C18TAB/MeO-Kaol_Wash (Figure 4(c,d)); the intercalation behaviors of C18TAC and C18TAB when applied to MeO-Kaol were different (Figure 1(c,e)). The basal spacing of MeO-Kaol increased from 0.86 to 1.12 nm upon the accommodation of methanol molecules [13], and so the MeO-Kaol used in this study was highly expanded by methanol in methanolic solutions containing C18TAC and C18TAB. In addition, the expansion relative to the original kaolinite layers was 0.40 nm (=1.12 nm − 0.72 nm). In contrast, it is well-known thata methanol molecules cannot intercalate into the original kaolinite layers [17]. Therefore, this 0.40 nm expansion increased the available reactive edges in the kaolinite. Because the plate-like kaolinite particles likely contained layers smaller than the lateral sizes of the particles [10], the edges on these layers may have become accessible following the expansion of the material. Further detailed studies will be required to assess the reactions of molecules having various sizes with kaolinites having differing interlayer distances, possibly using an anion-exchangeable kaolinite organic derivative [31]. Our group intends to undertake such studies in the future.

## 4. Conclusions

This work demonstrated increases in the number of available reactive edges in kaolinite samples following layer expansion, based on comparing the reactions of octadecyltrimethylammonium salts with kaolinite and MeO-Kaol. The present results provide an approach to assessing the contribution of edges depending on the degree of expansion of layered crystal materials.

## Data Availability

Available on request from the corresponding author.
